# First Complete Genome Sequence of a Chikungunya Virus Strain Isolated from a Patient Diagnosed with Dengue Virus Infection in Malaysia

**DOI:** 10.1128/genomeA.00876-16

**Published:** 2016-08-25

**Authors:** Man Kwan Ooi, Han Ming Gan, Ahmad Rohani, Sharifah Syed Hassan

**Affiliations:** a Virus-Host Interaction Research Group, Infectious Disease Laboratory, Jeffrey Cheah School of Medicine and Health Sciences, Monash University Malaysia, Jalan Lagoon Selatan, Bandar Sunway, Selangor, Malaysia; b Genomics Facility, Tropical Medicine and Biology Platform, Monash University Malaysia, Jalan Lagoon Selatan, Bandar Sunway, Selangor, Malaysia; c School of Science, Monash University Malaysia, Jalan Lagoon Selatan, Bandar Sunway, Selangor, Malaysia; d Medical Entomology Unit, Institute for Medical Research, Jalan Pahang, Kuala Lumpur, Malaysia

## Abstract

Here, we report the complete genome sequence of a chikungunya virus coinfection strain isolated from a dengue virus serotype 2-infected patient in Malaysia. This coinfection strain was determined to be of the Asian genotype and contains a novel insertion in the *nsP3* gene.

## GENOME ANNOUNCEMENT

Chikungunya virus (CHIKV) is an alphavirus in the family of *Togaviridae* and was first isolated in Tanzania in 1952 (1). CHIKV outbreaks in Africa and Asia have been reported since the 1960s, and reemergences of CHIKV have been observed (2). Global expansion of CHIKV outbreaks has broadened due to infected travelers, with the first known autochthonous CHIKV infection case in the Western Hemisphere reported in 2013 (3). Due to global expansion and reemergences of CHIKV outbreaks, CHIKV is recognized as an emerging epidemic-prone pathogen by the public health community (4). CHIKV infection has similar clinical manifestations to a few other mosquito-borne global health threat pathogens, particularly flaviviruses, which have a similar *Aedes* mosquito vector, e.g., dengue virus (DENV) (4). Due to the similar transmitting vector and geographical distribution of outbreaks, coinfection of DENV and CHIKV has emerged as a global threat, as coinfections usually remain undiagnosed (5, 6). Although coinfections of DENV and CHIKV were reported in India, Sri Lanka, Malaysia, Gabon, and Taiwan (7-12), to date, there is no complete genome sequence of CHIKV isolated from a coinfected patient with DENV. Here, we report the complete genome sequence of a CHIKV isolated from serum of a patient coinfected with DENV-2, in Selangor, Malaysia, in 2009.

Strain MUM001-2009-Selangor was isolated through *in vitro* limiting-dilution–plaque purification in Vero cells. The viral RNA was extracted from the virus supernatant using GENEzol reagent (Geneaid), and the cDNA was synthesized using Moloney murine leukemia virus (M-MLV) reverse transcriptase and random primers. Second-strand synthesis was performed using the NEB 2nd-strand synthesis kit (NEB, Ipswich, MA). The purified double-stranded DNA was subsequently prepped using Nextera XT (Illumina, San Diego, CA) and sequenced on the MiSeq (2 × 150 bp setting) located at the Monash University Malaysia Genomics Facility, Selangor, Malaysia. The draft genome was assembled using IDBA_UD (13) and subsequently gap filled using a conventional primer walking strategy.

The complete genome of CHIKV consists of 11,900 nucleotides (nt), containing two open reading frames (ORFs) coding for the typical structural and nonstructural proteins.

The preliminary phylogenetic analysis based on the neighbor-joining method showed that strain MUM001-2009-Selangor formed a monophyletic clustering (data not shown) with strains from the Asian genotype with close similarity (99%) strain MY/06/37348 (accession no. FN295483.3) and MY/06/37350 (accession no. FN295484.2) isolated from Malaysia**.** Intriguingly, a unique amino acid insertion in the *nsP3* gene was observed in this isolate in positions 376 to 451. This insertion consists of a duplicated sequence of part of the N-terminal domain of *nsP3*. Deletions of seven amino acids at this similar position starting from amino acid (aa) 376 were observed initially in a few of the Malaysian isolates, later in Indonesian isolates, and isolates currently circulating in the Western Hemisphere (14, 15). *nsP3* is involved in the negative-strand RNA synthesis, and the essential role remains enigmatic.

### Accession number(s).

The complete genome of coinfected chikungunya virus, Asian genotype, has been deposited in GenBank under the accession no. KX168429.

## References

[1] Lumsden WHR 1955 An epidemic of virus disease in southern Province, Tanganyika territory, in 1952-1953 II. General description and epidemiology. Trans R Soc Trop Med Hyg 49:33–57. doi:10.1016/0035-9203(55)90081-X. 14373835

[2] Powers AM, Logue CH 2007 Changing patterns of chikungunya virus: re-emergence of a zoonotic arbovirus. J Gen Virol 88:2363–2377. doi:10.1099/vir.0.82858-0. 17698645

[3] Nasci RS 2014 Movement of chikungunya virus into the Western Hemisphere. Emerg Infect Dis 20:1394–1395. doi:10.3201/eid2008.140333. 25061832 PMC4111178

[4] Staples JE, Breiman RF, Powers AM 2009 Chikungunya fever: an epidemiological review of a re-emerging infectious disease. Clin Infect Dis 49:942–948. doi:10.1086/605496. 19663604

[5] Caglioti C, Lalle E, Castilletti C, Carletti F, Capobianchi MR, Bordi L 2013 Chikungunya virus infection: an overview. New Microbiol 36:211–227. 23912863

[6] Carey DE 1971 Chikungunya and dengue: a case of mistaken identity? J Hist Med Allied Sci 26:243–262. doi:10.1093/jhmas/XXVI.3.243. 4938938

[7] Chahar HS, Bharaj P, Dar L, Guleria R, Kabra SK, Broor S 2009 Co-infections with chikungunya virus and dengue virus in Delhi, India. Emerg Infect Dis 15:1077–1080. doi:10.3201/eid1507.080638. 19624923 PMC2744227

[8] Chang S-F, Su C-L, Shu P-Y, Yang C-F, Liao T-L, Cheng C-H, Hu H-C, Huang J-H 2010 Concurrent isolation of chikungunya virus and dengue virus from a patient with coinfection resulting from a trip to Singapore. J Clin Microbiol 48:4586–4589. doi:10.1128/JCM.01228-10. 20881182 PMC3008485

[9] Hapaurachchi H, Bandara K, Hapugoda M, Williams S, Abeyewickreme W 2008 Laboratory confirmation of dengue and chikungunya co-infection. Ceylon Med J 53:104–105. doi:10.4038/cmj.v53i3.252. 18982804

[10] Leroy EM, Nkoghe D, Ollomo B, Nze-Nkogue C, Becquart P, Grard G, Pourrut X, Charrel R, Moureau G, Ndjoyi-Mbiguino A, De-Lamballerie X 2009 Concurrent chikungunya and dengue virus infections during simultaneous outbreaks, Gabon, 2007. Emerg Infect Dis 15:591–593. doi:10.3201/eid1504.080664. 19331740 PMC2671412

[11] Nayar SK, Noridah O, Paranthaman V, Ranjit K, Norizah I, Chem YK, Mustafa B, Chua KM 2007 Co-infection of dengue virus and chikungunya virus in two patients with acute febrile illness. Med J Malaysia 62:335–336. 18551940

[12] Schilling S, Emmerich P, Günther S, Schmidt-Chanasit J 2009 Dengue and chikungunya virus co-infection in a German traveller. J Clin Virol 45:163–164. doi:10.1016/j.jcv.2009.04.001. 19442576

[13] Peng Y, Leung HC, Yiu S-M, Chin FY 2010 IDBA–a practical iterative de Bruijn graph *de novo* assembler, p 426–440. In Berger B (ed), Research in computational molecular biology. Springer, New York, NY.

[14] Leparc-Goffart I, Nougairede A, Cassadou S, Prat C, De Lamballerie X 2014 Chikungunya in the Americas. Lancet 383:514. doi:10.1016/S0140-6736(14)60185-9. 24506907

[15] Sam I-C, Loong S-K, Michael JC, Chua C-L, Wan Sulaiman WY, Vythilingam I, Chan S-Y, Chiam C-W, Yeong Y-S, AbuBakar S, Chan YF 2012 Genotypic and phenotypic characterization of chikungunya virus of different genotypes from Malaysia. PLoS One 7:e50476. doi:10.1371/journal.pone.0050476. 23209750 PMC3507689

